# Predictive Value of Left Ventricular Systolic Dysfunction or Wall Motion Abnormalities for Non-Ischemic Myocardial Injury: A Multicenter Cardiovascular Resonance Study

**DOI:** 10.3390/jcm14113691

**Published:** 2025-05-24

**Authors:** Justyna M. Sokolska, Justyna Rajewska-Tabor, Marek Koziński, Dorota Kulawiak-Gałąska, Anna Jankowska, Małgorzata Pyda, Karol Miszalski-Jamka, Maciej Haberka

**Affiliations:** 1 Department of Cardiovascular Imaging, Faculty of Medicine, Institute of Heart Diseases, Wroclaw Medical University, 50-556 Wrocław, Poland; justyna.sokolska@umw.edu.pl; 2 Institute of Heart Diseases, Wroclaw University Hospital, 50-556 Wrocław, Poland; 3 1st Department of Cardiology, Poznan University of Medical Sciences, 61-848 Poznań, Poland; 4 Department of Cardiology, Kociewie Health Center, 83-200 Starogard Gdański, Poland; 5 1st Department of Cardiology, Medical University of Gdansk, 80-214 Gdańsk, Poland; 6 2nd Department of Radiology, Medical University of Gdańsk, 80-214 Gdańsk, Poland; 7 Department of Cardiology, Stefan Cardinal Wyszynski Regional Specialist Hospital, 20-718 Lublin, Poland; 8 Diagnostic Imaging Department, Silesian Center for Heart Diseases, 41-800 Zabrze, Poland; 9 Department of Cardiology, School of Health Sciences, Medical University of Silesia, 40-635 Katowice, Poland; 10 Department of Cardiovascular Imaging, Upper Silesian Medical Center of the Medical University of Silesia, 40-635 Katowice, Poland

**Keywords:** CMR, LGE, myocardial injury, systolic dysfunction, wall motion abnormalities

## Abstract

**Background:** Left ventricle (LV) systolic dysfunction, defined as a global (LVejection fraction, LVEF < 50%) and/or regional wall motion abnormalities (RWMA), are the major parameters assessed in patients with cardiovascular diseases. The study evaluated the predictive value of LV systolic dysfunction for non-ischemic myocardial injury (presence of myocardial fibrosis/scar) in patients with suspected myocarditis. **Methods**: This was a multicenter, observational, retrospective study (2018–2021) of stable outpatients with clinically suspected myocarditis referred for a contrast-enhanced CMR. Patients with a history of any other significant cardiovascular disorders were excluded from the study. In each patient, the LV systolic function (LVEF, RWMA) and the presence and severity of late gadolinium enhancement (LGE) were assessed by CMR. **Results**: A total of 773 consecutive patients were enrolled in the study. The average LVEF was 58 ± 10%, and systolic dysfunction was observed in 95 cases (12%). Subsequently, 456 patients (59%) with confirmed non-ischemic LGE in at least one segment were included in the study group. The average LVEF was 57 ± 11%, with LV systolic dysfunction observed in 126 (28%) individuals with RWMA and 84 (18%) with LVEF < 50%. The median number of LV segments with LGE was 3 (2–5), and the total amount of LGE was 6% (3–10) of the LV mass. The wall motion score index (WMSI) > 1 and LVEF < 56% were the best predictors of non-ischemic injury based on LGE (area under the curve [AUC] 0.62; sensitivity 31%; specificity 94%; *p* < 0.001 and AUC 0.59; sensitivity 42%; specificity 75%, *p* < 0.001, respectively). **Conclusions**: In stable patients with suspected myocarditis, any RWMA and LVEF < 56% had a predictive value for a non-ischemic myocardial injury as assessed by CMR.

## 1. Introduction

Global systolic function and regional wall motion of the left ventricle (LV) are among the most fundamental parameters assessed during routine echocardiographic examination [1]. Any abnormality in systolic function may provide significant diagnostic and prognostic information. It is well evidenced that patients with LV systolic dysfunction have a worse prognosis compared to those with normal systolic function [2,3]. Systolic dysfunction might be global, defined as lower LV ejection fraction (LVEF), and/or segmental, defined as regional wall motion abnormalities (RWMA) that are divided into hypokinesia, akinesia, or dyskinesia [1]. Reduced LVEF and/or RWMA require cardiac consultation and further investigations to determine the cause of the disorder and to make appropriate treatment decisions [3,4].

In patients with good acoustic windows, significant LV systolic dysfunction is easily detected in daily clinical practice with transthoracic echocardiography (TTE), which is even included in focused cardiac ultrasound—a first-line diagnostic tool for initial patient evaluation in acute settings [5]. However, in patients with poor acoustic windows due to obesity, chest and/or spine deformities, advanced chronic pulmonary diseases, or mild LV dysfunction, TTE may be challenging and has significant limitations in a reliable evaluation of systolic function [6]. Those limitations of TTE can be easily overcome with cardiovascular magnetic resonance (CMR), which is currently the gold standard for the precise assessment of LV systolic dysfunction in cardiac patients [6]. Moreover, CMR has a unique ability to assess tissue characteristics, including non-ischemic myocardial fibrosis and post-infarction scar, easily visualized by late gadolinium enhancement (LGE) after contrast administration [7,8].

Echocardiography is the first-line imaging method used in everyday clinical practice in patients suspected of myocarditis or LV injury. However, the real diagnostic value of LV normokinesis or LV systolic dysfunction in non-ischemic myocardial injury is still limited. Therefore, this study aimed to assess the predictive value of LV systolic dysfunction (LVEF < 50%) and RWMA, as assessed by CMR, for non-ischemic myocardial injury defined as myocarditis-like fibrosis (LGE) in patients referred for outpatient CMR due to clinical suspicion of myocarditis.

## 2. Materials and Methods

This was a multicenter, observational study with a retrospective analysis in 5 CMR centers. The consecutive stable outpatients referred for CMR due to cardiovascular symptoms and clinically suspected myocarditis (April 2018–October 2021) were screened for the eligibility criteria. Given the aim of the study, we decided to focus on myocarditis as a homogenous group of nonischemic injury.

The indication for CMR in all included patients was suspected myocarditis based on the clinical assessment of the referring physician. As the patients were stable, there were no indications for invasive endomyocardial biopsy as the first diagnostic tool to confirm myocarditis, but patients were sent for CMR to confirm non-ischemic causes of myocardial injury [9]. The exclusion criteria were as follows: (1) a history of myocardial infarction or previous myocarditis; (2) other cardiovascular disease, including significant valve diseases, cardiomyopathy, congenital heart diseases, or previous cardiac surgery; (3) suboptimal CMR image quality due to the patient’s noncompliance or arrhythmia.

The concomitant diseases (diabetes, dyslipidemia, hypertension, obesity, chronic kidney disease) were reported in the clinical characteristics based on the prior diagnosis and/or current treatment [10,11,12].

### 2.1. Cardiovascular Magnetic Resonance Imaging

All the CMR images were obtained on the 1.5T systems: GE Optima MR450w (GE Healthcare, Waukesha, WI, USA), Magnetom Aera (Siemens, Erlangen, Germany), Magnetom Avanto (Siemens, Erlangen, Germany) with a dedicated phased-array cardiac coil or body matrix coil. All CMR examinations were ECG-gated and performed according to the routine clinical protocols in line with current guidelines [13,14]. The CMR protocol was described in our previous paper [15]. In brief, the following sequences were performed: multi-planar SSFP acquisitions for functional sequences in short- and long-axis, T2-weighted edema imaging with fat saturation sequences, and LGE sequences for fibrosis evaluation performed 10–15 min after contrast infusion (0.1 mmol/kg of body weight of gadobutrol, Gadovist 1.0, Bayer Inc., Mississauga, ON, Canada). LGE acquisitions were based on the same planes as the short- and long-axis SSFP images: transverse short-axis LV planes and longitudinal LV planes.

The CMR scans were obtained and analyzed by experienced research teams in 5 centers with a long-standing practice in CMR (8–20 years). Cardiac volumes, mass, and function (LV and right ventricle (RV) end-diastolic and end-systolic volumes; ejection fraction (EF)) were analyzed using dedicated commercial software (CVI42, Circle Cardiovascular Imaging Inc., Calgary, AB, Canada). All volumes and LV mass were indexed to body surface area (BSA) and were interpreted using gender- and age-adjusted reference values provided by the European Association of Cardiovascular Imaging guidelines [16,17].

The LV myocardium was segmented according to the American Heart Association 17-segment model [18]. LV segmental function was assessed using a 4-point scale: normal (1 point), hypokinetic (2 points), akinetic (3 points), or dyskinetic (4 points). The wall motion score index (WMSI) [19] was then calculated as the sum of all segment scores divided by 17.

Myocarditis-like injury was defined as non-ischemic mid-wall and/or subepicardial LGE [13,14]. All segments were evaluated for the presence and the severity of LGE by the semiquantitative method, which was calculated as the ratio of LGE area to the segment area. Afterward, the values (%) for each segment were summed up in total LGE values expressed as ratio (%) and mass (g).

### 2.2. Statistical Analysis

The distribution of variables was tested for normality with the Kolmogorov–Smirnov test. Numerical variables were presented as means with standard deviations (SD) or medians with interquartile ranges (IQR), as appropriate. Categorical variables were expressed as numbers and percentages. Baseline clinical characteristics were compared between subgroups using the unpaired Student’s *t*-test for normally distributed continuous variables or the Mann–Whitney U test for non-normal distributions. The differences between the proportions were analyzed using the Chi-squared test. Associations between numerical variables were assessed using Pearson or Spearman correlation coefficients. The receiver operating characteristic (ROC) curve analysis was used to determine optimal cut-off values of the baseline clinical parameters for the prediction of myocardial injury or dysfunction. A *p*-value < 0.05 was considered statistically significant. All analyses were performed using Medcalc software (version 19.1, Ostend, Belgium).

## 3. Results

A total of 773 consecutive patients with suspected myocarditis scheduled for CMR were enrolled in the study. The average age of patients was 44 ± 14 years old and 47% were female. The mean LVEF was 58 ± 10%, and systolic dysfunction was observed in 94 (12%) patients with LVEF < 50% and 138 (18%) patients with RWMA. Detailed clinical and CMR characteristics of all patients with suspected myocarditis enrolled in the study are presented in Table 1.

**Figure 1:**
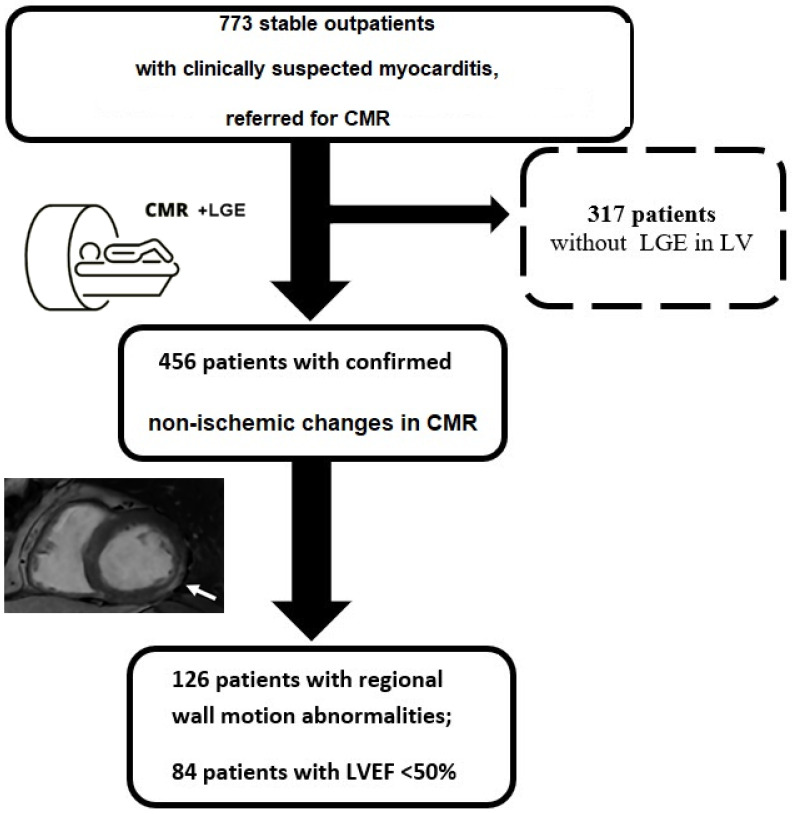
The study flowchart presents the inclusion and exclusion criteria for patients with suspected myocarditis.

Afterward, 456 patients (59%) with confirmed presence of non-ischemic LGE in at least one LV segment were included in the study group (Figure 1—study flowchart). The average LVEF was 57 ± 11%, with 126 (28%) individuals with RWMA and 84 (18%) with LVEF < 50%.

**Figure 2:**
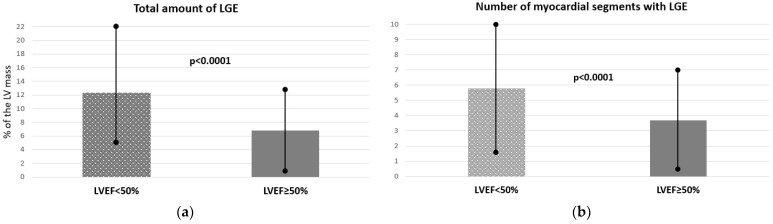
Total amount of late gadolinium enhancement (LGE) in patients with confirmed non-ischemic changes in cardiac magnetic resonance according to their global systolic function: (**a**) total amount of LGE presented in percentage of the left ventricle total mass; (**b**) number of myocardial segments with LGE. Abbreviations: LGE, late gadolinium enhancement; LVEF, left ventricular ejection fraction. Results are presented as mean ± standard deviation.

All patients with RWMA had only hypokinesia, and no akinesia or dyskinesia were observed. The median number of LV segments with LGE was 3 [2,3,4,5], and the total amount of LGE was 6% of the LV mass. The most common locations of LGE were basal inferior (36%), inferoseptal (29%), and inferolateral (27%) segments of the LV. Detailed clinical and CMR characteristics of patients with non-ischemic injury in CMR are presented in Table 2. The study group was divided into two subgroups based on LV function. Patients with confirmed myocarditis-like injury and LVEF < 50% were significantly older and had more LGE (see Figure 2a) and more myocardial segments with LGE (see Figure 2b) compared to patients with preserved LVEF. Detailed clinical and CMR characteristics of patients with non-ischemic injury in CMR, divided according to LVEF, are presented in Table 3.

Furthermore, the predictive values toward non-ischemic LGE were performed in all the subjects (n = 773). Global systolic dysfunction, defined as the LVEF < 50%, failed to show a predictive value for LGE in patients with suspected myocarditis (area under the curve [AUC] 0.527; sensitivity 19%; specificity 96%; positive predictive value [PPV] 88; negative predictive value [NPV] 45; *p* = 0.2). However, in the ROC curve analysis, the WMSI > 1 and LVEF < 56% were the predictors of myocarditis-like LGE (AUC 0.62; sensitivity 31%; specificity 94%; PPV 88; NPV 48.5 and AUC 0.59; sensitivity 42%; specificity 75%; PPV 70; NPV 47, all *p* < 0.001, respectively).

Moreover, the WMSI showed a positive association (r = 0.3, *p* < 0.0001) and the LVEF showed a negative association (r = −0.2, *p* = 0.0001) with the total percentage of LGE.

## 4. Discussion

In this large multicenter, retrospective study, which assessed stable outpatients with clinically suspected myocarditis, CMR confirmed the initial diagnosis in more than half of the cases. The major finding was that RWMA and/or LVEF < 56% assessed in CMR had a predictive value for the presence of non-ischemic myocardial injury (LGE). The specificity of RWMA was quite high, whereas for LVEF < 56% it was only moderate. However, the sensitivity of both predictors was low, even when assessed in high-quality images obtained via CMR, which highlights the limitation of relying solely on systolic function for identifying myocarditis-like myocardial fibrosis. Given the limitations of TTE, and as shown in our study population, preserved LVEF and normal regional wall motion do not exclude non-ischemic myocardial injury. Therefore, all patients with suspected myocarditis should be scheduled for the reference method of comprehensive CMR with LGE and, optimally, also T1 and T2 mapping.

In 1985, Medina et al. tested the predictive value of RWMA assessed by echocardiography in detecting coronary artery disease (CAD) in patients with normal and dilated LV [20]. They found that RWMA was highly suggestive of significant CAD [19]. Later, the worsening wall motion in the echocardiographic dobutamine test was shown to provide prognostic information on cardiac events in patients with known and suspected CAD [21]. However, much less is known about underlying myocardial injury, especially with a non-ischemic pattern, in the presence of systolic dysfunction [22,23,24,25,26,27]. In our study, only one-third of patients with a non-ischemic LGE pattern had RWMA, and only one-fifth had decreased LVEF < 50%. This confirms the limited diagnostic utility of functional parameters, such as RWMA and decreased LVEF, in detecting non-ischemic myocardial injury.

Contrast-enhanced CMR is a well-known, valuable diagnostic workup method for visualizing myocardial injury in patients with ischemic and non-ischemic diseases, such as myocarditis and cardiomyopathies [7,14]. Different patterns of LGE allow for easy distinction between ischemic and non-ischemic myocardial fibrosis or scars, whereas simple functional imaging cannot provide any reliable information about the etiology of myocardial dysfunction [28]. Subendocardial or transmural LGE presented in a specific coronary artery territory usually indicates an infarct scar, which is strongly correlated with irreversible ischemic injury and ventricular remodeling [29,30]. In contrast, LGE that appears in the midwall or epicardial layer of the myocardium is diagnostic for non-ischemic myocardial injury seen in different types of cardiomyopathies and myocarditis [13,14,29]. Non-ischemic LGE may represent a combination of necrosis and fibrosis, as well as ongoing inflammation, especially when other tissue characteristics in T2-weighted images or T2 mapping show myocardial edema [13]. Therefore, in myocarditis, the extent and even the presence of LGE may vary depending on the time between acute myocarditis and the CMR study [31,32,33].

Importantly, identifying any LGE as a presence of myocardial injury, regardless of etiology, is not only diagnostically relevant but also raises crucial considerations for risk stratification [34,35]. In ischemic heart disease, a greater extent of LGE was associated with major cardiac events, such as hospitalization for heart failure, whereas the presence of transmural scar was correlated with the risk of arrhythmias and responses to cardiac resynchronization therapy [33,36]. Similarly, non-ischemic LGE has also been associated with worse outcomes, including increased risk of arrhythmias and heart failure progression [37,38,39]. Myocarditis is a common cause of cardiac problems, especially in a younger population [40]. In our study group, non-ischemic changes were confirmed in approximately 60% of stable outpatients referred for CMR due to clinical suspicion of myocarditis.

Therefore, more advanced imaging techniques are necessary for correct diagnosis, especially in patients with clinical symptoms, normal LVEF, and no RWMA. CMR with contrast and LGE sequences may provide tissue characterization and myocardial injury. It is an important diagnostic tool in stable patients with suspected myocarditis, who may avoid invasive endomyocardial biopsy [9,41,42].

### Limitation of the Study

Our study was based on a retrospective analysis of stable outpatients. The study population did not include unstable patients with acute myocarditis. Therefore, the predictive value of LV dysfunction or RWMA in acute myocardial injury may be different. Moreover, we did not include a T1 or T2 mapping, as those sequences were not available in all centers at the time of recruitment. According to the current guidelines and reflecting daily clinical practice [9], as the studied population was clinically stable, no endomyocardial biopsies were performed for histopathological confirmation of myocarditis; therefore, no information regarding the etiology of non-ischemic myocardial injury was available.

## 5. Conclusions

RWMA and/or LVEF < 56% assessed via CMR had a predictive value with moderate to high specificity, but low sensitivity, for detecting non-ischemic myocardial injury defined as the presence of a non-ischemic LGE pattern. Therefore, assessment of systolic function alone—whether by echocardiography or CMR—may be insufficient in patients with suspected non-ischemic injury, and comprehensive contrast-enhanced CMR with tissue characterization should be considered to improve diagnostic accuracy in this population.

## Figures and Tables

**Table 1:** Clinical characteristics and cardiac magnetic resonance findings in all outpatients with suspected myocarditis.

	Patients with Suspected Myocarditis n = 773
Age, years	44 ± 14
Female, n (%)	366 (47)
Hypertension, n (%)	210 (27)
Diabetes, n (%)	50 (6)
Hypercholesterolemia, n (%)	110 (14)
Coronary artery disease, n (%)	36 (5)
Body mass index, kg/m^2^	26.8 ± 4.8
Obesity, n (%)	174 (22)
**CMR findings**	
LVEDV, mL	159 ± 49
LVEF, %	58 ± 10
LVEF < 50%, n (%)	95 (12)
LV mass, g	120 ± 35
Regional wall motion abnormalities, n (%)	138 (18)
RVEDV, mL	143 ± 40
RVEF, %	55 ± 8

Abbreviations: CMR, cardiac magnetic resonance; LV, left ventricle; LVEDV, left ventricular end-diastolic volume; LVEF, left ventricular ejection fraction; RVEDV, right ventricular end-diastolic volume; RVEF, right ventricular ejection fraction. Results are presented as the number of patients (and percentage), mean ± standard deviation, or median (with lower and upper quantiles).

**Table 2:** Clinical characteristics and cardiac magnetic resonance findings in patients with confirmed non-ischemic injury in cardiac magnetic resonance.

	Patients with Confirmed Non-Ischemic Injury in CMR n = 456
Age, years	43 ± 14.5
Female, n (%)	177 (39)
Hypertension, n (%)	124 (27)
Diabetes, n (%)	38 (8)
Hypercholesterolemia, n (%)	53 (12)
Coronary artery disease, n (%)	29 (6)
Body mass index, kg/m^2^	26.9 ± 4.8
Obesity, n (%)	100 (22)
**CMR findings**	
LVEDV, mL	165 ± 55
LVEF, %	57 ± 11
LVEF < 50%, n (%)	84 (18)
LV mass, g	127 ± 37
Regional wall motion abnormalities, n (%)	126 (28)
Number of myocardial segments with LGE	3 (25–75)
Number of patients with LGE in 1–3 myocardial segments, n (%)	258 (57)
Total amount of LGE in % of the LV mass	5.9 (2.9–10.3)
Total amount of LGE, g	9.9 (4.9–17.3)
RVEDV, mL	146 ± 41
RVEF, %	55 ± 9

Abbreviations: CMR, cardiac magnetic resonance; LGE, late gadolinium enhancement; LV, left ventricle; LVEDV, left ventricular end-diastolic volume; LVEF, left ventricular ejection fraction; RVEDV, right ventricular end-diastolic volume; RVEF, right ventricular ejection fraction. Results are presented as the number of patients (and percentage), mean ± standard deviation, or median (with lower and upper quantiles).

**Table 3:** Clinical characteristics and cardiac magnetic resonance findings in patients with non-ischemic injury in CMR, divided according to LVEF.

	Patients with LVEF ≥ 50% n = 372	Patients with LVEF < 50% n = 84	*p*
Age, years	42 ± 14	46 ± 16	0.03
Female, n (%)	151 (41)	26 (31)	0.1
Hypertension, n (%)	92 (24)	32 (38)	0.08
Diabetes, n (%)	24 (6)	14 (16)	0.0001
Hypercholesterolemia, n (%)	40 (11)	13 (15)	0.26
Coronary artery disease, n (%)	22 (6)	7 (8)	0.47
Body mass index, kg/m^2^	27 ± 5	28 ± 5	0.03
Obesity, n (%)	76 (20)	24 (28)	0.1
**CMR findings**			
LVEDV, mL	152 ± 38	223 ± 80	<0.0001
LVEF, %	60 ± 6	39 ± 9	<0.0001
LV mass, g	121 ± 31	158 ± 49	<0.0001
WMSI	1.03 ± 0.07	1.59 ± 0.48	<0.0001
Number of myocardial segments with LGE	4 ± 3	6 ± 4	<0.0001
Total amount of LGE in % of the LV mass	7 ± 6	12 ± 10	<0.0001
RVEDV, mL	143 ± 39	158 ± 48	0.004
RVEF, %	56 ± 8	49 ± 11	<0.0001

Abbreviations: CMR, cardiac magnetic resonance; LGE, late gadolinium enhancement; LV, left ventricle; LVEDV, left ventricular end-diastolic volume; LVEF, left ventricular ejection fraction; RVEDV, right ventricular end-diastolic volume; RVEF, right ventricular ejection fraction; WMSI, wall motion score index. Results are presented as the number of patients (and percentage), mean ± standard deviation, or median (with lower and upper quantiles).

## Data Availability

The data underlying this article will be shared upon reasonable request to the corresponding author.

## Abbreviations

The following abbreviations are used in this manuscript:

AUC	area under the curve
BSA	body surface area
CAD	coronary artery disease
CMR	cardiac magnetic resonance
ECG	electrocardiogram
EF	ejection fraction
IQR	interquartile range
LGE	late gadolinium enhancement
LV	left ventricle
LVEF	left ventricular ejection fraction
LVEDV	left ventricular end-diastolic volume
NPV	negative predictive value
TTE	transthoracic echocardiography
PPV	positive predictive value
ROC	receiver operating characteristic
RWMA	regional wall motion abnormalities
RV	right ventricle
RVEDV	right ventricular end-diastolic volume
RVEF	right ventricular ejection fraction
SD	standard deviation
SSFP	steady-state free precession
WMSI	wall motion score index
